# Representing logic gates over Euclidean space via heaviside step function

**DOI:** 10.1038/s41598-022-11941-y

**Published:** 2022-05-14

**Authors:** Giovanni Iacovelli, Claudio Iacovelli

**Affiliations:** 1 Department of Electrical and Information Engineering (DEI), Politecnico di Bari, 70126 Bari, Italy; 2 Consorzio Nazionale Interuniversitario per le Telecomunicazioni (CNIT), 43124 Parma, Italy; 3 Institut de Ciències Fotòniques (ICFO), The Barcelona Institute of Science and Technology, 08860 Castelldefels, Spain

**Keywords:** Applied mathematics, Computer science, Information technology, Engineering

## Abstract

Theoretical concepts asserted by Alan Turing are the basis of the computation and hence of machine intelligence. Turing Machine, the fundamental computational model, has been proven to be reducible to a logic circuit and, at the same time, portable into a computer program that can be expressed through a combination of fundamental programming language control structures. This work proposes a mathematical framework that analytically models logic gates employing Heaviside Step Function. The existence of a correspondence between a generic finite-time algorithm and the proposed mathematical formulation is proven. The proposed interpretation is given through a well-defined logical circuit analytical expression. Relevant geometrical applications, related to polygon processing, having wide implications in engineering branches are presented together with a new Penalty Method for constrained optimization problems handling. A detailed simulation campaign is conducted to assess the effectiveness of the applications derived from the proposed mathematical framework.

## Introduction

The concept of algorithm^1^ was first introduced when David Hilbert posed the *Entscheidungsproblem* in 1928. Still, it does not have a unique definition. Essentially, it can be regarded as a finite succession of elementary instructions which leads to a result in a finite amount of time. The first and most famous mathematical interpretation was proposed by Kurt Gödel in the early ’30s who introduced the notion of recursive functions^2^. Algorithm first characterization took place in 1936 when Alan Turing created a theoretical model, called Turing Machine (TM)^3^ in order to prove that Hilbert’s problem was unsolvable.

Under specific conditions, any function computed by TM, deterministic or not, can be reduced to a circuit which is composed by a set of Logic Gates (LGs)^4^. Such digital devices implement a Boolean function that takes one or more binary inputs and provides a binary output. Further, patches of circuits can be synthetized into a complicated mathematical objects. For instance, this could either be the transfer function of the electronic circuit or an object that is associated to the truth table of it. Those objects can be inputs of other gates whose definition can be extended to accept and return not only numbers but also functions. The composition of this type of gates results in the description of the whole circuit. In mathematical terms, binary status can be expressed and LGs can be realized as results of specific composition of a certain number of Heaviside Step Functions (HSFs).

This idea can also be applied to programming languages, because they provide an high-level abstraction of the machine language that is mapped into logic circuits. In fact, Böhm–Jacopini’s Theorem^5^ states that (i) every algorithm/function can be implemented through sequence, selection and iteration (ii) every TM, up to an extent, is equivalent to a program with only compositions and iterations. This means that it is possible to associate an algorithm to a specific problem, a TM to the algorithm, a logic circuit to the TM and a function to the logic circuit. Therefore, the main goal of this article is to correlate an analytical function to a given algorithm. In fact, it will be shown how the HSF is sufficient to represent the fundamental control structures and to analytically model the LGs.

Within this framework, it is possible to reformulate analytical/geometrical problems which pertain to computer graphics, computer vision, robotics and geoscience. In these fields, one of the fundamental topics is polygons processing which is examined in several studies^6–9^ in literature. In^10^, two algorithms are designed to discover intersection points between simple polygons. The first is a set-based intersection algorithm while the second is binary-search-tree-based algorithm which allows the authors to briefly describe a set of possible merging and Boolean operations among polygons. These are also the focus of^11^ where the proposed algorithm is also capable to deal with polygons with holes. In^12^ proposes a GPU-based rasterized polygon intersection area algorithm able to work with complex and large-scale ones. Further, in^13^ an algorithm capable of performing clipping, Boolean union and difference between input polygons is presented. Based on entry/exit intersection point property, it uses an efficient data structure and can easily also be adapted to Boolean operations between regions composed by input sets. These kind of geometrical problems have also implications in medical imaging. For instance, in^14^ a method is developed for the analytical calculation of the 3D shape and volume of volumes’ intersection in order to reconstruct 3D Positron Emission Tomography data. In Robotics geometrical information about configuration^15^, placement, collision^16^ or proximity^17^ among objects are major concerns. In^18^, an algorithm is proposed in order to avoid collisions between a manipulator and an obstacle. These are represented by cylinders and convex plane polygons, respectively, while the intersection among these describes the physical contact. In^19^, an algorithm is designed to address another challenging aspect: motion planning. This work takes into account planar polygonal rigid robots that have to move in a polyhedral environment and avoid collisions thanks to decomposability property. The process of determining the robot’s position and the obstacles places involves Point-in-Polygon Problem. In^20^, instead, the authors propose an algorithm capable of solving this issue and deal with concave and convex polygons of different shapes. Throughout the article, a subset of these applications will be recast as optimization problems, via the conceived framework.

Summing up, the contributions of this work are:

- The correspondence between a finite-time algorithm and its analytical form, by means of the proposed framework, is proved. In particular, a definition of the LGs over Euclidean space is given through the HSF, namely HSFG. Further, for the sake of applicability, an equivalent smoothed form of the obtained results is also presented by employing the logistic function. An interpretation of the fundamental control structures, i.e., sequence, selection, and iteration, is also given.
- Geometrical problems previously described, together with some other geometrical applications, are tackled from an analytical perspective, thus avoiding the development of specific algorithms. Specifically, computation of volume of intersecting loci is deeply investigated to demonstrate how the mathematical construction naturally leads back to the HSFGs employment.
- A new Penalty Method to handle non-convex and even constrained optimization problems is studied. The resulting cost function requires to be probed by means of optimization techniques^21^ or Artificial Intelligence Search Algorithms (SAs)^22^ which are able to pick the optimum or quasi-optimum solution in the output space. Intersection points finding between two implicit curves is proposed as concrete geometrical application.

Without loss of generality, Genetic Algorithm (GA)^23^ is used in all the investigated applications which require optimization. Nevertheless, arbitrary SA can be adopted since the proposed framework is orthogonal with respect to the algorithm used. Moreover, since the problems are handled from an analytical perspective, a direct comparison with other algorithmic solutions is not in the scope of this work. Nonetheless, conducted numerical simulations thoroughly evaluate the aforementioned contributions, in order to prove the effectiveness of the proposal. To the best of authors’ knowledge, such results have never been retrieved before.

The present work is organized as follows: “HSF Boolean logic” introduces mathematical definitions and properties of LGs realized through HSF. “Continuous HSF circuit definition” discusses continuous representation of HSF adopted. “HSP control structures” establishes a relation between algorithms and proposed gates. With “Geometrical applications”, formulation of the solutions to a set of envisioned problems is given. Section “HSF penalty method”, instead, presents a new Penalty Method for constrained optimization problems based on examined notions. Section “Numerical evaluation”, analyses numerical results derived from theoretical concepts discussed. Finally, “Conclusion” concludes the work and draws future works and research perspectives.

## HSF Boolean logic

In this Section, a mathematical characterization is addressed to achieve a proper definition of LGs functions realized by $\Theta (x)$, i.e HSF. Information is carried by *x*, and its binary description is achieved through a discrimination with respect to a threshold, i.e. zero value. In the most complicated case $\Theta (x)$ could not be defined for $x=0$. On the contrary, the upcoming concepts will result in a exemplification.

### HSF circuit definition

Without loss of generality, let the possible inputs be described with a vector space over a real scalar field, i.e. ${\mathbb {R}}^D$, and $O=\{\mathbf{x }\in {\mathbb {R}}^D|\exists \ j:1...D \ni ' x_j=0\}$. Therefore, the definition of the function that encodes the real inputs in binary information is:

1 $$\begin{aligned}&\vec {\Theta }_0:{\mathbb {R}}^D\backslash O\rightarrow {\mathcal {B}}^D,\nonumber \\&\mathbf{x }\rightarrow \vec {\Theta }_0(\mathbf{x }):=\left( \Theta (x_1),...,\Theta (x_D)\right) ^T, \end{aligned}$$

where ${\mathcal {B}} = \{0,1\}$. Let $R\ge 1\in {\mathbb {N}}$, $N_1=D$ and $N_{R+1}=Q$. Then, it is necessary to define the following sets as:

$$\begin{aligned}&\{N_r|r:1...R+1,N_r\in {\mathbb {N}}\},\\&\{\vec g_r:{\mathcal {B}}^{N_r}\rightarrow {\mathbb {R}}^{N_{r+1}}\}_{r:1...R},\\&\{\vec \Theta _{r}:{\mathbb {R}}^{N_{r+1}}\backslash O_{r+1}\rightarrow {\mathcal {B}}^{N_{r+1}}\}_{r:1...R}, \end{aligned}$$

where $O_{r+1}$ is the analogous of *O* for any arbitrary input $\mathbf{x }\in {\mathbb {R}}^{N_{r+1}}$, $\vec \Theta _r$ acts in the same manner as $\vec \Theta _0$, and *R* is the rank, i.e. the number of compositions the HSF Circuit (HSFC) will be made of. Moreover, $\vec g_r$, *r* : 1...*R* must be uniform continuous functions. In presence of a chain of compositions, the inner argument of $\vec \Theta _{r+1}$ together with its kernel is:

2 $$\begin{aligned}{}&\vec h_{r+1}=\vec g_{r+1}\circ \vec \Theta _{r}\circ \vec h_r,\ r:1..R-1,\ h_1=\vec g_1, \nonumber \\&K(\vec h_{r})=\{\vec \Theta _0(\mathbf{x })\in {\mathcal {B}}^D|\ \exists i:1...N_{r+1}\ni ' h_{r}^i(\vec \Theta _0(\mathbf{x }))=0\}. \end{aligned}$$

These kernels have to be excluded, because $\vec \Theta _{r}(\vec h_{r}(\vec \Theta _0(\mathbf{x })\in \mathrm {Ker}(h_{r}))$ would not be defined. Obviously, the only argument that has to be checked is $h_1$ if the rank is *R* = 1. Therefore, if ${\mathcal {K}}$ is the union of all kernels, the proposed definition of a HSFC ${\mathcal {H}}$ of rank *R* is:

3 $$\begin{aligned}&{\mathcal {H}}^R:{\mathcal {B}}^D\backslash {\mathcal {K}}\rightarrow {\mathcal {B}}^Q, \end{aligned}$$

4 $$\begin{aligned}&\mathbf{y }\rightarrow {\mathcal {H}}^R(\mathbf{y })=\Big (\bigcirc _{r=1}^R\vec \Theta _r\circ \vec g_r\Big )(\mathbf{y }), \end{aligned}$$

where $\bigcirc _{r=1}^R(\cdot ):=\vec \Theta _R\circ \vec g_R\circ ...\circ \vec \Theta _1\circ \vec g_1$, and $\mathbf{y }=\vec \Theta _0(\mathbf{x })$ is the input string. If $\Theta (0)$ is defined, then the previous definition also holds in ${\mathcal {K}}$. Indeed, this is a Boolean function with input $\vec \Theta _0\big (\mathbf{x }\big )$. The set of these functions does not have a group structure. However, the set is closed with respect to the composition operation. In the spirit of this notation, the most trivial circuit is the identity which is obtained by setting *R* = 1 and *D* = *Q*:

5 $$\begin{aligned}&{\mathcal {I}}:{\mathcal {B}}^D\rightarrow {\mathcal {B}}^D,\nonumber \\&\mathbf{y }\rightarrow \vec \Theta _0(\mathbf{y }-{\varvec{\xi }})=\mathbf{y } , \end{aligned}$$

derived from Eq. (4) with:

6 $$\begin{aligned}&\vec g_1:{\mathcal {B}}^D\rightarrow {\mathbb {R}}^D, \ \mathbf{y }\rightarrow \mathbf{y }-{\varvec{\xi }},\nonumber \\&\vec \Theta _1:{\mathbb {R}}^D\backslash {\mathcal {K}} \rightarrow {\mathcal {B}}^D, \ \mathbf{z }\rightarrow \vec \Theta _0(\mathbf{z }), \end{aligned}$$

where ${\varvec{\xi }}\in ]0,1[^D$ being an arbitrary parameter which takes into account the fact that $\Theta (0)$ may not be defined or may be different from zero. This translation allows to have ${\mathcal {K}} = K(h_1) = K(\mathbf{y }-{\varvec{\xi }}) = \emptyset$.

#### Remark 1

Similar considerations can be done for any set of functions $\vec f_i:{\mathbb {R}}^{N_{i+1}}\rightarrow {\mathcal {B}}^{N_{i+1}} \ i:0...R$, that encodes in binary the information, by substituting $\vec \Theta _i(\cdot )$ in the chain of composition. Indeed, $\vec \Theta _i(\cdot )$ can be another HSFC.

### Fundamental HSF gates

It is worth defining an equivalent LG standard basis $\Psi _L=\{NOT,AND,OR\}$ with the use of Eq. (4). The HSF representation of $\Psi _L$ is defined as the unitary rank (*R* = 1) basis $\Psi _H=\{N,\Lambda ,\Omega \}$, constituted by the fundamental HSFGs. These particular circuits are:

- NOT:
  $$\begin{aligned}&\vec g_1:{\mathcal {B}}^D\rightarrow {\mathbb {R}}^D, \quad \quad \ \mathbf{y }\rightarrow \vec g_1(\mathbf{y }) = (\mathbf{1 }-\mathbf{y })-{\varvec{\xi }},\\&\vec \Theta _1:{\mathbb {R}}^D\rightarrow {\mathcal {B}}^D, \quad \quad \ \mathbf{z }\rightarrow \vec \Theta _0(\mathbf{z }),\\&\vec \Theta _1\circ g_1:{\mathcal {B}}^D\rightarrow {\mathcal {B}}^D, \ \mathbf{y }\rightarrow \vec \Theta _0((\mathbf{1 }-\mathbf{y })-{\varvec{\xi }}) = {\mathcal {I}}(\mathbf{1 }-\mathbf{y }), \end{aligned}$$
  7 $$\begin{aligned} \vec N(\mathbf{x }):=\mathbf{1 }-\vec \Theta (\mathbf{x })=\vec \Theta (-\mathbf{x }), \end{aligned}$$
  with $${\varvec{\xi }}\in ]0,1[^D$$.
- AND:
  $$\begin{aligned}&g_1:{\mathcal {B}}^D\rightarrow {\mathbb {R}}, \quad \quad \ \mathbf{y }\rightarrow g_1(\mathbf{y }) = \prod _{i=1}^D y_i-\xi ,\\&\Theta _1:{\mathbb {R}}\rightarrow {\mathcal {B}}, \quad \quad \ z\rightarrow \Theta (z),\\&\Theta _1\circ g_1:{\mathcal {B}}^D\rightarrow {\mathcal {B}}, \ \mathbf{y }\rightarrow \Theta (\prod _{i=1}^D y_i-\xi ) = {\mathcal {I}}(\prod _{i=1}^D y_i), \end{aligned}$$
  8 $$\begin{aligned} \Lambda (\mathbf{x }):=\prod _{i=1}^D\Theta (x_i). \end{aligned}$$
- OR:
  $$\begin{aligned}&g_1:{\mathcal {B}}^D\rightarrow {\mathbb {R}}, \quad \quad \ \mathbf{y }\rightarrow g_1(\mathbf{y }) = \sum _{i=1}^D y_i-\xi , \\&\Theta _1:{\mathbb {R}}\rightarrow {\mathcal {B}}, \quad \quad \ z\rightarrow \Theta (z),\\&\Theta _1\circ g_1:{\mathcal {B}}^D\rightarrow {\mathcal {B}}, \ \mathbf{y }\rightarrow \Theta (\sum _{i=1}^D y_i-\xi ), \end{aligned}$$
  9 $$\begin{aligned} \Omega (\mathbf{x }):=\Theta (\sum _{i=1}^D\Theta (x_i)-\xi ), \end{aligned}$$
  with $$\xi \in ]0,1[$$.

As a matter of fact, for a given binary input $\mathbf{y }$, the output of any HSFG of $\Psi _H$ is the same of its correspondent LG in $\Psi _L$. Again, the absence of a definition of $\Theta (0)$ is taken into account thanks to $\xi$.

#### Theorem 1

Let **y**$\in {\mathcal {B}}^D\backslash {\mathcal {K}}$ be the input of a HSFG ${\mathcal {H}}^R$. Then, there must exist a logic circuit *C* such that $C(\mathbf{y })={\mathcal {H}}^R(\mathbf{y })$ where $C(\mathbf{y })$ is the output of *C* receiving **y**.

#### Proof

According to the definition of $\Psi _H$, each function of this set has an I/O relation which has an exact correspondence with the truth table of its analogous in $\Psi _L$. Since $\Psi _L$ can reproduce any truth table of any logic circuit *C* via composition of its LGs, then the same must hold for $\Psi _H$. This means that any HSFG ${\mathcal {H}}^R$ determines an I/O relation which must be reproducible through a composition of functions in $\Psi _L$. $\square$

#### Corollary 1

NAND is capable of constructing any HSFC via compositions, because it must be as fundamental as $\Psi _H$. The same also holds for NOR. Recalling Remark 1, these can be obtained by composing elements from the $\Psi _H$ basis, in the following manner:

- NAND $${\overline{\Lambda }}$$:= $$N \circ \Lambda$$
  10 $$\begin{aligned} {\overline{\Lambda }}(\mathbf{x }):=1 - \prod _{i=1}^D\Theta (x_i). \end{aligned}$$
- NOR $${\overline{\Omega }}$$:= $$N \circ \Omega$$
  11 $$\begin{aligned} {\overline{\Omega }}(\mathbf{x }):=\Theta (\xi - \sum _{i=1}^D\Theta (x_i)), \end{aligned}$$
  with $$\xi \in ]0,1[$$.

#### Corollary 2

DeMorgan’s Theorems are applicable to HSFGs which compose the basis $\Psi _H$.

12 $$\begin{aligned} \Lambda \circ \vec N=N\circ \Omega , \quad \Omega \circ \vec N=N\circ \Lambda . \end{aligned}$$

## Continuous HSF circuit definition

**Figure 1:**
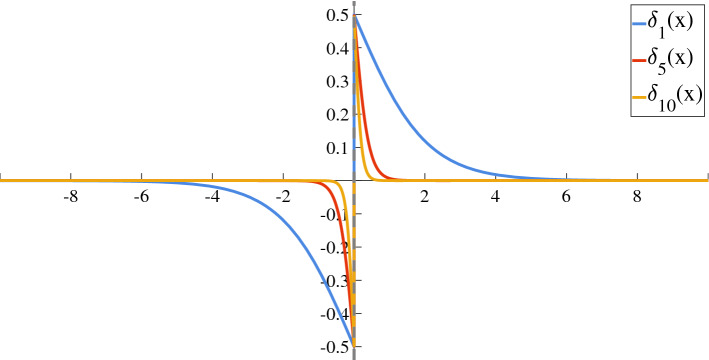
$\delta _\alpha (x)$ for different values of $\alpha$.

The HSF is not suited for contexts and applications in which the differentiability of the functions is required. Several continuous approximations are available but, in this article, the proposed substitution is:

13 $$\begin{aligned} \Theta (x) \rightarrow \sigma (\alpha x)\equiv \sigma _\alpha (x):=\frac{1}{1+e^{-\alpha x}}, \end{aligned}$$

where $\alpha$ is a coefficient that should be set sufficiently high and $\sigma$ is the Sigmoid Function (SF), which has been widely used in engineering and physics applications^24,25^. The difference between the two functions, depicted in Fig. 1, is defined as:

14 $$\begin{aligned} \delta _\alpha (x) := \Theta (x) - \sigma (\alpha x) = {\left\{ \begin{array}{ll} \frac{e^{-\alpha x}}{1+e^{-\alpha x}} \quad &{}\mathrm {if} \ x>0\\ -\frac{1}{1+e^{-\alpha x}} \quad &{}\mathrm {if} \ x<0\\ \vartheta _0-\frac{1}{2} \quad &{}\mathrm {if} \ x=0 \end{array}\right. }, \end{aligned}$$

where $\vartheta _0=\Theta (0)$. It can be compactly reformulated as

15 $$\begin{aligned} \delta _\alpha (x) = \frac{e^{-\alpha x}}{1+e^{-\alpha x}}\Theta (x) - \frac{1}{1+e^{-\alpha x}}\Theta (-x), \end{aligned}$$

$\delta _{0}=\delta _\alpha (0)$ is not taken into account, since in this formulation $\delta _0=0$, independently of $\vartheta _0$. $\delta _\alpha$ is an odd function in ${\mathbb {R}}^*$ and it is odd in ${\mathbb {R}}$ only if $\delta _0=0$: this means that (15) is odd in ${\mathbb {R}}$. Moreover, $\delta _\alpha$ goes exponentially to zero $\forall x\ne 0$ if $\alpha \rightarrow \infty$. At the same time, it goes exponentially to zero $\forall \alpha >0$ when $x\rightarrow \pm \infty$ and its 1-norm is $\frac{2\ln (2)}{\alpha }$. Therefore, the difference between $\Theta (\cdot )$ and $\sigma (\alpha \cdot )$ is small if $\alpha$ is large.

The continuous version of a circuit, namely SF Circuit (SFC), is now seen as a function of a real input:

16 $$\begin{aligned}&{\mathcal {H}}^R_\alpha :\widetilde{{\mathcal {B}}}^D\rightarrow \widetilde{{\mathcal {B}}}^Q\nonumber \\&\mathbf{y }\rightarrow {\mathcal {H}}_\alpha ^R(\mathbf{y })=\left( \bigcirc _{r=1}^R\vec \sigma _{r,\alpha }\circ \vec g_r\right) (\mathbf{y }), \end{aligned}$$

where $$\widetilde{{\mathcal {B}}} = ]0,1[$$, $$\mathbf{y } = \big (\vec \sigma _{0,\alpha }(\mathbf{x })\big )$$,

17 $$\begin{aligned}&\vec \sigma _{0,\alpha }(\mathbf{x }):{\mathbb {R}}^D\rightarrow \widetilde{{\mathcal {B}}}^D\nonumber \\&\mathbf{x }\rightarrow \vec \sigma _{0,\alpha }(\mathbf{x })=(\sigma _\alpha (x_1),...,\sigma _\alpha (x_D))^T, \end{aligned}$$

and $\{\vec g_r:[0,1]^{N_r}\rightarrow {\mathbb {R}}^{N_{r+1}}\}_r$ is a set of uniformly continuous functions. Since the logistic function is defined $\forall x \in {\mathbb {R}}$, the circuit can clearly accept any $\mathbf{x }\in {\mathbb {R}}^D$. Until the end of this Section, the following will be considered as functions of $\mathbf{x }$, i.e. ${\mathcal {H}}^R(\mathbf{x })\equiv \big ({\mathcal {H}}^R\circ \vec \Theta _0\big )(\mathbf{x })$, ${\mathcal {H}}_\alpha ^R\equiv \big ({\mathcal {H}}_\alpha ^R\circ \vec \sigma _{0,\alpha }\big )(\mathbf{x })$.

The uniform convergence of a SFC to its correspondent HSFCs can be proven, provided that some considerations are taken into account. The function $\vec \Theta _0(\mathbf{x })$ splits the domain in $Q_k$ D-cubes, with $k:1...2^D$, and it assumes a constant vector value within each $Q^*_k=Q_k\backslash O$. Now let *R* be the rank of a given circuit ${\mathcal {H}}^R$. Since any composition (which ${\mathcal {H}}^R$ is made of) factorizes the domain no further than the action of $\vec \Theta _0$, and since any inner argument of the composition is a constant within the reduced D-cube $Q^*_k$, then $\forall r \ \vec h_r(\mathbf{x })\equiv \big (\vec h_r\circ \vec \Theta _0\big )(\mathbf{x })=\vec c_{r,k}\ \forall \mathbf{x }\in Q^*_k$, i.e. $\forall r\ \vec h_r\big |_{Q^*_k}= \vec c_{r,k}$. However, to have a proper definition of $\vec h_r$ in $Q^*_k$, it must be that $\forall q:1..r-1:\ h_q^u(\mathbf{x })=c^u_{q,k}\ne 0\ \forall u:1..N_{q+1}$, with *u* labelling the components of the vector $\vec h_r$. Otherwise, the action of $\vec \Theta _q$ is not defined in $Q^*_k$, implying $dom({\mathcal {H}}^R)\cap Q^*_k = \emptyset$. Therefore, if the previous happens $\forall k:1..2^D$, it must be concluded that $dom({\mathcal {H}}^R)=\emptyset$. This whole argument is an equivalent picture in the input domain of the kernels defined in (2). Now, let $X_t = \{\mathbf{x } \in {\mathbb {R}}^D \ni ' |x_j|\ge t \ \forall j:1...D\}$ with $t>0$ and define $X_{t,k} := X_t \cap Q_k$.

### Theorem 2

Let $\{\vec g_r\}_r$ be a set of uniformly continuous functions, $J = \{k:1...2^D|\ \forall \ r:1...R, \forall u:1..N_{r+1}:\ h_r^u\big |_{Q_k} = c^u_{r,k} \ne 0\}$, ${\mathcal {H}}^R$ a HSFC and ${\mathcal {H}}_\alpha ^R$ a SFC. Then, ${\mathcal {H}}_\alpha ^R$ converges in

$$\begin{aligned}&{\widetilde{X}} := \bigcup _{k \in J} X_{t,k}, \end{aligned}$$

uniformly to ${\mathcal {H}}^R$ for $\alpha \rightarrow \infty$.

### Proof

See **Appendix A**. $$\square$$

Moreover, to get an estimation of the error committed in the evaluation of ${\mathcal {H}}^R_\alpha (\mathbf{x })$ in place of a given ${\mathcal {H}}^R(\mathbf{x })$, the circuits may be regarded as functionals with respect to either $\sigma _\alpha$ or $\Theta$. As a consequence, the notation ${\mathcal {H}}[\Theta ](\mathbf{x })$ describes Eq. (4) while ${\mathcal {H}}[\sigma _\alpha ](\mathbf{x })$ describes Eq. (16). Now, the equality ${\mathcal {H}}^R[\Theta ]={\mathcal {H}}^R[\sigma _\alpha +\delta _\alpha ](\mathbf{x })$ is trivial, and the variational Taylor series reads:

18 $$\begin{aligned} {\mathcal {H}}^R[\Theta ]&={\mathcal {H}}^R[\sigma _\alpha +\epsilon \delta _\alpha ]\Big |_{\epsilon =1} \nonumber \\&={\mathcal {H}}^R[\sigma _\alpha ]+\sum _{k=1}^{\infty }\frac{d^k}{d\epsilon ^k}{\mathcal {H}}^R[\sigma _\alpha +\epsilon \delta _\alpha ]\Big |_{\epsilon =0}\frac{\epsilon ^k}{k!}\ \ @\epsilon =1. \end{aligned}$$

This series is convergent, and it collects both the error and the discontinuity in the functional derivatives. In the case of interest, the parameter $\epsilon$ is set to one for the equality to hold, but the variation $\delta _\alpha$ has been proved to be small, so it is expected that the majority of the discrepancy has to be caused by the first orders. Finally, thanks to this analysis, it is possible to define $\Psi _\sigma$ basis which is equivalent to $\Psi _H$ but exploiting the logistic function. The fundamental SF Gates (SFGs) that constitute this new basis are $N_\alpha ,\Lambda _\alpha ,\Omega _\alpha$ for which Corollary 2 still holds.

## HSP control structures

Once defined the analytical structure of a general SFG, a correlation can be established between them and algorithms.

John E. Savage proved the following, which appears as Theorem 3.8.1 of^4^.

### Theorem 3

Any computation performed by a one-tape Turing machine M, deterministic or nondeterministic, on an input string **w** in T steps using m b-bit memory cells can be simulated by a circuit ${\mathcal {C}}_{M,T}$ over the standard complete basis $\Omega$ of size and depth O(ST) and O(T log S), respectively, where S = mb is the storage capacity in bits of M’s tape. For the deterministic TM the inputs to this circuit consist of the values of **w**. For the nondeterministic TM the inputs consist of **w** and the Boolean choice input variables whose values are not set in advance.

Based on the above Theorem, the following is proved.

### Theorem 4

Let ${\mathcal {P}}$ be a program or an algorithm which computes a function in a finite amount of time. Then, there must exist an equivalent HSFG ${\mathcal {H}}$.

### Proof

Recalling Böhm–Jacopini’s Theorem, ${\mathcal {P}}$ is equivalent to TM *M* and can be expressed through a combination of the three fundamental control structures: sequence, selection and iteration. Therefore, reminding Theorem 3 it is possible to state that there exists a logic circuit *C* equivalent to *M*. Finally, considering Theorem 1 can be claimed that there must exist a HSFC ${\mathcal {H}}$ which is equivalent to *C*. Hence, control structures must also have a correspondent expression in these terms. $\square$

A slight interpretation is presented hereby. However, it does not pretend to be mathematically rigorous as it will considered for future investigations. The proposed characterization is proved to be sufficient for the description of control structures which compose a finite-time algorithm. For this reason, the states of all variables and operations can be expressed through Boolean strings and HSFCs, respectively. This means that a set of commands is translated into a set of HSFCs $\{{\mathcal {H}}^{R_r}_r\}_{r:1...S}$, and to execute this set in sequence means:

19 $$\begin{aligned} \mathbf{x }_{OUT} = \big (\bigcirc _{r=1}^S {\mathcal {H}}^{R_r}_r\big )(\mathbf{x }_{IN}), \end{aligned}$$

where $\mathbf{x }_{OUT}$ and $\mathbf{x }_{IN}$ are the state variable before and after the sequence.

The selection, instead, is inherently addressed by HSF. In fact, given logical conditions which have to be verified, this control structure can be described through a HSFC ${\mathcal {H}}^R$. This returns 1 if the conditions are not satisfied and 0 otherwise. The mathematical expression is:

20 $$\begin{aligned} \mathbf{x }_{OUT} = \big (f_1{\mathcal {H}}^R+f_0(1-{\mathcal {H}}^R)\big )(\mathbf{x }_{IN}), \end{aligned}$$

where $f_1$ and $f_0$ are the statements executed in false and true cases, respectively.

Finally, for what concerns finite iteration, the definition of a recursive function turns to be necessary:

$$\begin{aligned}&\mathbf{x }_1 = \mathbf{x }_{IN}{\mathcal {H}}^R(\mathbf{x }_{IN})+f(\mathbf{x }_{IN})(1-{\mathcal {H}}^R(\mathbf{x }_{IN}))\\&\mathbf{x }_2 = \mathbf{x }_1{\mathcal {H}}^R(\mathbf{x }_1)+f(\mathbf{x }_1)(1-{\mathcal {H}}^R(\mathbf{x }_1))\\&\vdots \\&\mathbf{x }_{n} = \mathbf{x }_{n-1}{\mathcal {H}}^R(\mathbf{x }_{n-1})+f(\mathbf{x }_{n-1})(1-{\mathcal {H}}^R(\mathbf{x }_{n-1})). \end{aligned}$$

This synthetizes into

21 $$\begin{aligned} \mathbf{x }_n&=\bigcirc _{i=1}^{n-1}f(\mathbf{x }_{IN}){\mathcal {H}}^R\left( \bigcirc _{i=1}^{n-1}f(\mathbf{x }_{IN})\right) \nonumber \\&+\left( 1-{\mathcal {H}}^R\left( \bigcirc _{i=1}^{n-1}f(\mathbf{x }_{IN})\right) \right) \bigcirc _{i=1}^{n}f(\mathbf{x }_{IN}). \end{aligned}$$

In fact, once the *n*-th step is reached, all the others have to be

22 $$\begin{aligned} \mathbf{x }_k=f(\mathbf{x }_{k-1})=\bigcirc _{i=1}^{k-1}f(\mathbf{x }_{IN}), \quad \forall k:1...n-1. \end{aligned}$$

If the iteration ends at the $n+1$-th step, then the output is:

23 $$\begin{aligned} \mathbf{x }_{n+1}=\mathbf{x }_n=\bigcirc _{i=1}^nf(\mathbf{x }_{IN}), \end{aligned}$$

and this happens if the following condition is satisfied:

24 $$\begin{aligned} \forall \mathbf{x }_{IN}\in {\mathcal {L}}\ \exists \ n\in {\mathbb {N}}\ni '{\mathcal {H}}^R\big (\bigcirc _{i=1}^{n-1}f(\mathbf{x }_{IN})\big )=1, \end{aligned}$$

with ${\mathcal {L}}\subseteq {\mathcal {B}}^D$ being the domain of the inputs.

## Geometrical applications

In this Section, a set of geometrical applications derived from previous theoretical discussion are deeply investigated.

### Computation of volume measure covered by intersecting geometrical loci

The first application studied enables the computation of the volume of the union of different loci. The whole mathematical process is deeply described hereby and it is concluded with the equivalent interpretation in terms of HSFGs.

Consider a finite set of geometrical loci, described with

25 $$\begin{aligned} \big \{f_n(\mathbf{x },\mathbf{q }_n) = 0 \big | n:1..N\big \}, \end{aligned}$$

where $\mathbf{x }$ is the generic coordinate in the space ${\mathbb {R}}^D$ in which the loci are embedded and $\mathbf{q }_n$ is a point in the the vector space of parameters which specifies the locus. Addressing the point $\mathbf{q }_n$ with index *n* is equal to consider such a point and its dimensionality to be independent from all the others. It is also assumed that $f_n$ identifies a closed and compact volume

26 $$\begin{aligned} V_n=\Big \{\mathbf{x }\Big |f_n(\mathbf{x },\mathbf{q }_n) \le 0\Big \}. \end{aligned}$$

The aim is to find an expression which describes the volume that is effectively encompassed by the set of loci, namely

27 $$\begin{aligned} V= \bigcup _{n=1}^NV_n=\Big \{\mathbf{x }\Big |\bigvee _{n=1}^N f_n(\mathbf{x },\mathbf{q }_n) \le 0\Big \}. \end{aligned}$$

Clearly, the major difficulty stands in the fact that the sum of the measures

28 $$\begin{aligned} \mu _S = \sum _n \mu \big (V_n\big ) =\sum _n\int _{V_n}d\mathbf{x }, \end{aligned}$$

counts the intersections among different $V_n$ a certain number of times, which will be later studied. Obviously, in the trivial case in which no intersections are present

29 $$\begin{aligned} \mu _S = \mu \big (V\big ) \iff V_i\ \cap \ V_j=\emptyset \ \forall i,j:1..N,\ i \ne j. \end{aligned}$$

**Figure 2:**
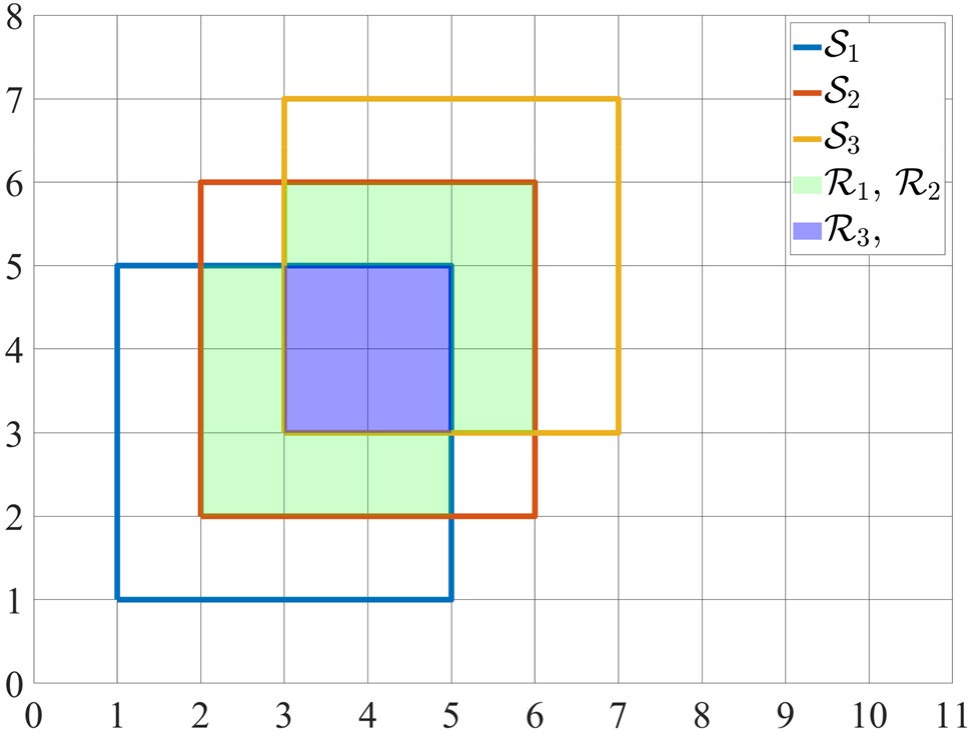
Example scenario of intersecting squares.

Otherwise, in order to obtain $\mu \big (V\big )$ from $\mu _S$, each intersecting region has to be subtracted in an appropriate way. In other words, intersections have to be counted just once. For instance, consider a *D* = 2 scenario in which three squares ${\mathcal {S}}_i$, *i* : 1..3 intersect in region ${\mathcal {R}}_i$, *i* : 1..3 as shown in Fig. 2. Assuming *v* as the measure of the unit square, $\mu _S$ is equal to 48*v*, while $\mu \big ( V \big )$ = 30*v*. Defining ${\mathcal {R}}_{1} = {\mathcal {S}}_1\cap {\mathcal {S}}_2$, ${\mathcal {R}}_{2} = {\mathcal {S}}_2\cap {\mathcal {S}}_3$ and ${\mathcal {R}}_3 = {\mathcal {S}}_1\cap {\mathcal {S}}_2\cap {\mathcal {S}}_3$, it results (in *v* units) that

30 $$\begin{aligned} \mu \big (V\big )&=\mu _S - \mu \big ({\mathcal {R}}_{1}) - \mu \big ({\mathcal {R}}_{2}\big ) - 2\mu \big ({\mathcal {R}}_{3}\big )\nonumber \\&=48-5-5-2\times 4=30. \end{aligned}$$

As previously stated, the measure of each intersection region has to be subtracted a certain number of times that is equal to their multiplicity $\kappa$. But first, the characteristic function of the *n*-th volume has to be introduced:

31 $$\begin{aligned} {\mathcal {X}}\Big [f_n\Big ](\mathbf{x }):=\Theta \big (-f_n(\mathbf{x },\mathbf{q }_n)\big ){\left\{ \begin{array}{ll}1\ \ \ \ \ \mathrm {if}\ f_n(\mathbf{x },\mathbf{q }_n)<0\\ 0\ \ \ \ \ \mathrm {if}\ f_n(\mathbf{x },\mathbf{q }_n)>0\\ \vartheta _0\ \ \ \mathrm {if}\ f_n(\mathbf{x },\mathbf{q }_n)=0.\end{array}\right. } \end{aligned}$$

According to the definition of the Heaviside used in this formula, the points verifying $f_n = 0$ count as $\vartheta _0$ that is usually equal to 1 or 1/2, but they also may be not counted at all if $\vartheta _0$ is 0 or NaN. Then, the multiplicity of a point $\mathbf{x }$ is defined as follows:

32 $$\begin{aligned} \kappa (\mathbf{x }):=\sum _n{\mathcal {X}}\Big [f_n\Big ](\mathbf{x })-1. \end{aligned}$$

In particular, the multiplicity of a point $\mathbf{x }^*$ which belongs just to one volume is $\kappa (\mathbf{x }^*)=0$

.

Consider a subspace ${\mathcal {M}} \subseteq {\mathbb {R}}^D \ni ' V_n \subseteq {\mathcal {M}} \ \forall n$. Analytically, the contribution that has to be subtracted from (28) is

33 $$\begin{aligned} \int _{\mathcal {M}}\kappa (\mathbf{x })d\mathbf{x }. \end{aligned}$$

However, an issue emerges in this formulation since

$$\begin{aligned} \forall \mathbf{x }\in {\mathcal {M}}\ni '\ \mathbf{x }\notin \ V: \kappa (\mathbf{x })=-1. \end{aligned}$$

In fact, each point of ${\mathcal {M}}$ that does not belong to any $V_n$ has -1 multiplicity, thus incorrectly increasing $\mu _S$. A third addendum which rectifies this behaviour is needed:

34 $$\begin{aligned} \int _{\mathcal {M}} \Theta \bigg (\xi -\sum _n {\mathcal {X}}\Big [f_n\Big ](\mathbf{x })\bigg ) d\mathbf{x }. \end{aligned}$$

As a matter of fact, the integrand is equal to $1 \ \forall \mathbf{x } \ni ' f_n > 0 \ \forall n$. The parameter $\xi$ is a shift that imposes the aforementioned behaviour. Indeed, points which do not belong to any volume return a value which is different from 0 according to the adopted definition of Heaviside:

35 $$\begin{aligned} \xi \in {\left\{ \begin{array}{ll} {]}0,1[ &{}\text {if}\, \vartheta _0 = 1\\ {]}0,1/2[ &{}\text {if}\, \vartheta _0 = 1/2\\ {[}0,1[ &{}\text {if}\, \vartheta _0 = 0 \end{array}\right. } \end{aligned}$$

Basically, as the results in (28)(33)(34) are brought together:

36 $$\begin{aligned} \mu \big (V\big ) = \mu _S - \int _{\mathcal {M}}\kappa (\mathbf{x })d\mathbf{x } - \int _{\mathcal {M}} \Theta \bigg (\xi -\sum _n {\mathcal {X}}\Big [f_n\Big ](\mathbf{x })\bigg ) d\mathbf{x }. \end{aligned}$$

The expression above can be rewritten in a more compact manner. It is worth noting that (33) is equivalent to

37 $$\begin{aligned} \sum _n\int _{\mathcal {M}} {\mathcal {X}}\Big [f_n\Big ](\mathbf{x }) d\mathbf{x } - \int _{\mathcal {M}}d\mathbf{x } = \sum _n\int _{V_n}d\mathbf{x } - \int _{\mathcal {M}}d\mathbf{x }. \end{aligned}$$

The exchange between the sum and integral signs can be always done if *N* is finite, and also for $N \rightarrow \infty$ under the hypothesis of Fubini’s Theorem. Moreover, the first integrand is equal to 1 for every point belonging to $V_n$ at fixed *n* and it is 0 elsewhere. Therefore, the whole first addendum is equivalent to (28). Hence, (36) becomes:

38 $$\begin{aligned} \int _{\mathcal {M}} \bigg (1 - \Theta \Big (\xi -\sum _n {\mathcal {X}}\Big [f_n\Big ](\mathbf{x })\Big )\bigg ) d\mathbf{x }. \end{aligned}$$

Recalling results achieved in “HSF Boolean logic”, it is clear that (38) is the following HSFGs composition:

39 $$\begin{aligned} \Big (N \circ {\overline{\Omega }} \circ \vec N\Big )(f_n(\mathbf{q }_n))_{n}(\mathbf{x }) = \Big (\Omega \circ \vec N\Big )(f_n(\mathbf{q }_n))_{n}(\mathbf{x }), \end{aligned}$$

which does not correspond to any element of the $\Psi _H$ basis. However, it can be described by Algorithm 1 thanks to Theorem 4. Finally, this leads to:

40 $$\begin{aligned} \mu \big (V\big ) {:}{=} {\mathcal {V}}&= \int _{\mathcal {M}} \Omega \Big [\big \{N(f_n)\big \}\Big ](\mathbf{x }) d\mathbf{x } \end{aligned}$$

41 $$\begin{aligned}&= \int _{\mathcal {M}}\Theta \Big (\sum _n {\mathcal {X}}\Big [f_n\Big ](\mathbf{x }) - \xi \Big )d\mathbf{x }, \end{aligned}$$

which perfectly describes (27). This expression, as stated in Corollary 2, is also equivalent to

42 $$\begin{aligned} {\mathcal {V}} = \int _{\mathcal {M}} {\overline{\Lambda }}\Big [\big \{f_n\big \}\Big ](\mathbf{x }) d\mathbf{x }. \end{aligned}$$

#### Remark 2

The characteristic function may not be defined on the locus $f_n=0$ due to the definition of $\vartheta _0$. Nonetheless, the measure of a volume does not depend on the value assumed by $\vartheta _0$ because such value concerns the border which is a zero measure set.

#### Remark 3

It is worth noting that is sufficient to change the sign of the argument of all HSFs in Eq. (41) to characterize the dual formula for the computation of the intersection among all volumes, that is:

43 $$\begin{aligned} V^*= & {} \bigcap _{n=1}^NV_n=\Big \{\mathbf{x }\Big |\bigwedge _{n=1}^N f_n(\mathbf{x },\mathbf{q }_n) \le 0\Big \}. \end{aligned}$$

44 $$\begin{aligned} \mu \big (V^*\big ):={\mathcal {V}}^*= & {} \int _{\mathcal {M}} \Theta \Big (\xi - \sum _n \Theta \big (f_n(\mathbf{x },\mathbf{q }_n) \big )\Big ) d\mathbf{x }\nonumber \\= & {} \int _{\mathcal {M}} {\overline{\Omega }}\Big [\big \{f_n\big \}\Big ](\mathbf{x }) d\mathbf{x }. \end{aligned}$$

Again, recalling Corollary 2, an equivalent expression is

45 $$\begin{aligned} {\mathcal {V}}^* = \int _{\mathcal {M}} \Lambda \Big [\big \{N(f_n)\big \}\Big ](\mathbf{x }) d\mathbf{x }, \end{aligned}$$

that well describes (43).


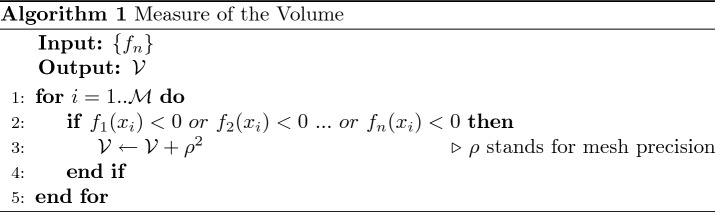


#### Error analysis

The error which is introduced by the use of the logistic function in place of Heaviside is addressed hereby. First, it is necessary to introduce the expression of the error:

46 $$\begin{aligned} \delta {\mathcal {V}} := \int _{\mathcal {M}} \bigg [&\Theta \Big (\sum _n \Theta \big (-f_n(\mathbf{x },\mathbf{q }_n) \big ) - \xi \Big ) \nonumber \\&- \sigma \Big (\alpha \Big (\sum _n \sigma \big (-\alpha f_n(\mathbf{x },\mathbf{q }_n) \big )- \xi \Big )\Big )\bigg ] d\mathbf{x } . \end{aligned}$$

Considering (18), introduce the variable $z=\alpha \sum _n(\sigma _\alpha +\epsilon \delta _\alpha )(-f_n)-\xi$ and consider that any term $\frac{d^k}{d\epsilon ^k}z$, $k\ge 2$ is zero, therefore it will not be written. As the same time, it can be reasonably assumed that the perturbation is zero at the border and, as a consequence, terms which are border-related are zero too.

Hence, it can be written:

47 $$\begin{aligned}\frac{d}{d\epsilon }{\mathcal {V}}&=\int d\mathbf{x }\bigg (\frac{d}{dz}\sigma _\alpha (z)\frac{d}{d\epsilon }z+\delta _\alpha (z)+\epsilon \frac{d}{dz}\delta _\alpha (z)\frac{d}{d\epsilon }z\bigg )\nonumber ,\\ \frac{d^2}{d\epsilon ^2}{\mathcal {V}}&=\int d\mathbf{x }\bigg (\frac{d^2}{dz^2}\sigma _\alpha (z)\left( \frac{d}{d\epsilon }z\right) ^2+2\frac{d}{dz}\delta _\alpha (z)\frac{d}{d\epsilon }z \nonumber \\&\quad +\frac{d^2}{dz^2}\delta _\alpha (z)\left( \frac{d}{d\epsilon }z\right) ^2\bigg )\nonumber ,\\ \frac{d^3}{d\epsilon ^3}{\mathcal {V}}&=\int d\mathbf{x }\bigg (\frac{d^3}{dz^3}\sigma _\alpha (z)\left( \frac{d}{d\epsilon }z\right) ^3+2\frac{d^2}{dz^2}\delta _\alpha (z)\left( \frac{d}{d\epsilon }z\right) ^2 \nonumber \\&\quad +\frac{d^3}{dz^3}\delta _\alpha (z)\left( \frac{d}{d\epsilon }z\right) ^3\bigg )\nonumber ,\\ \frac{d^k}{d\epsilon ^k}{\mathcal {V}}&=\int d\mathbf{x }\bigg (\frac{d^k}{dz^k}\sigma _\alpha (z)\left( \frac{d}{d\epsilon }z\right) ^k \nonumber \\ &\quad +2\frac{d^{k-1}}{dz^{k-1}}\delta _\alpha (z)\left( \frac{d}{d\epsilon }z\right) ^{k-1}+\frac{d^k}{dz^k}\delta _\alpha (z)\left( \frac{d}{d\epsilon }z\right) ^k\bigg ). \end{aligned}$$

Moreover, $\frac{d^n}{dz^n}\delta _\alpha (z)=-\frac{d^n}{dz^n}\sigma _\alpha (z)$ because of the definition of $\delta _\alpha$, so the first and third addenda for $k\ge 2$ simplify at every term. Let $z'$ be equal to $z(\epsilon =0)=\sum _n\sigma _\alpha (-f_n)-\xi$ and manipulate the following:

48 $$\begin{aligned} \frac{d}{d\epsilon }{\mathcal {V}}+\frac{d^2}{d\epsilon ^2}{\mathcal {V}}\frac{1}{2}\bigg |_{\epsilon =0}=\int d\mathbf{x }\bigg (\delta _{\alpha }(z)+2\frac{d}{dz}\delta _{\alpha }(z)\frac{d}{d\epsilon }z\bigg ). \end{aligned}$$

Finally, as $\frac{d}{d\epsilon }z=\alpha \sum _n\delta (-f_n)$ and the index of the Taylor series is moved back, it results that

49 $$\begin{aligned} \delta {\mathcal {V}}=\delta _\alpha (z')-2\sum _{j=1}^\infty \frac{\sigma _\alpha ^{(j)}(z')}{j!}\Big (\alpha \sum _n\delta _\alpha (-f_n)\Big )^j. \end{aligned}$$

### Custom D-dimensional volumes

Another interesting application of Heaviside is the possibility to represent customizable volumes. In fact, the technique presented in previous Section is not limited just to analytical functions. Without loss of generality, consider $D = 2$. Suppose that this desired area is defined by a closed polygonal chain of *N* segments, each of them lying on a given line described by $f_n$ (or the vertical line $x = c$, where *c* is a constant value). Clearly, $\Theta (f_n)$ is a binary representation of the inequality $f_n \ge 0$, because the equation $\Theta (f_n) = 1$ is satisfied only in a subspace of ${\mathbb {R}}^2$. Capitalizing the concept of AND HSFG described in “HSF Boolean logic”, the customizable area is cast with the following characteristic function:

50 $$\begin{aligned} {\mathcal {X}}_\Theta = \prod _{n=1}^N \Theta (f_n) = \Lambda \Big [\big \{f_n\big \}\Big ] . \end{aligned}$$

This concept does not rely on choice of $f_n$ as these can be anything, as long as ${\mathcal {X}}_\Theta = 1$ encompasses a non-zero finite area. It is worth noting that with this formulation the Point-in-Polygon problem is inherently addressed by evaluating (50) in the desired location.

#### Remark 4

Reminding results of “Continuous HSF circuit definition”, also for this application is possible to define an equivalent SF form ${\mathcal {X}}_\sigma$, taking advantage of $\Lambda _\sigma$.

#### Remark 5

It should be noticed that

51 $$\begin{aligned} \Omega (\{N(f_n)\}_n,\{\Lambda _q(\{f_m\}_{m(q)})\}_q)\quad \forall n,q,m(q), \end{aligned}$$

represents a general partition of input domain into a certain number of disjointed sub-domains of arbitrary shape.

### Custom D-dimensional borders

According to the formulation proposed so far, a further important result is achieved when the objective is not to calculate the volume but to have an expression of its border. Once again, it is sufficient to exploit the AND HSFG. It is necessary that $\vartheta _0 \ne 0$ to distinguish the locus of interest in the following manner:

52 $$\begin{aligned} \Gamma :\ \Theta ({\mathcal {Y}}_\Theta - \vartheta _0)\Theta (\vartheta _0 - {\mathcal {Y}}_\Theta ) - \vartheta _0^2 = 0 , \end{aligned}$$

where ${\mathcal {Y}}_\Theta$ is the generic characteristic function which can be composed in one of the several ways previously discussed. In practise, when two face-to-face Heavisides are multiplied, the only geometrical locus that has non-zero value is the desired border. In other words, an equality constraint is imposed as will be seen in the next Section. In particular, if $\vartheta _0 \ne \{0,1\}$ then it is just needed:

53 $$\begin{aligned} \Gamma :\ \Theta ({\mathcal {Y}}_\Theta - \vartheta _0) - \vartheta _0 = 0 . \end{aligned}$$

However, it must be noticed that if ${\mathcal {Y}}_\Theta$ is composed of more than one function, and their borders intersect, then Eqs. (52) and (53) are not satisfied at the intersections of borders, except for Eq. (52) when $\vartheta _0 = 1$.

Another way to realize this locus is the substitution defined in (13) so that previous formulation is equivalent to:

54 $$\begin{aligned} \Gamma :\ \lim _{\alpha \rightarrow \infty }\sigma \big (\alpha ({\mathcal {Y}}_\sigma - 1/2)\big ) - 1/2 = 0 , \end{aligned}$$

where ${\mathcal {Y}}_\sigma$ is the characteristic function expressed via SF. Still, intersections do not belong to $\Gamma$. If the limit is not taken, an interesting behaviour arises in the proximity of intersections due to the analyticity of $\sigma$:

55 $$\begin{aligned} \Gamma _\alpha :\ \sigma \big (\alpha ({\mathcal {Y}}_\sigma - 1/2)\big ) - 1/2 = 0 . \end{aligned}$$

**Figure 3:**
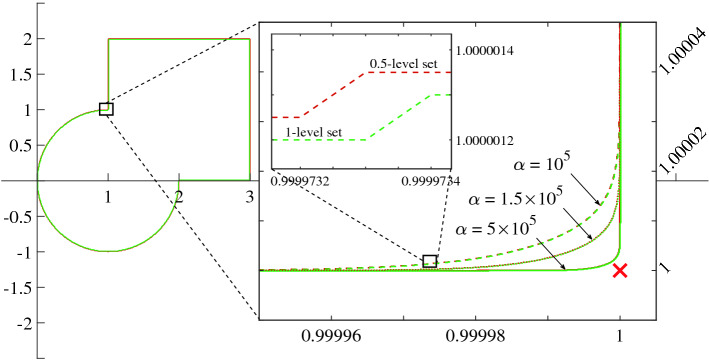
A custom border as function of $\alpha$.

For instance, consider the configuration in Fig. 3. It consists of a square centered in $[2 \ 2]^T$ and a circle in $[1 \ 0]^T$ which intersect in $[1\ 1]^T$. Therefore, the ${\mathcal {Y}}_\sigma$ in Eq. (55) is the sum of the characteristic functions of the square and the circle. The former is made with Eq. (50) and the usual substitution (13), while the latter is done with Eq. (31). As $\alpha$ increases, $\Gamma _\alpha$ gets closer to the real intersection. The presence of the error owed to $\alpha$ results to be negligible, and even advantageous: the proposed formulation represents an optimum analytical approximation of the union border, which in principle (i.e. the exact border) should have no defined gradient at the intersection points.

## HSF penalty method

In this Section, an alternative representation of constrained-optimization problems is proposed. This is possible thanks to binary logic granted by HSF. Let us have a cost function ${\mathcal {A}}(\mathbf{q })$ that has to be minimized and it is subject to some equality and inequality constraints

$$\begin{aligned}&\min {\mathcal {A}}(\mathbf{q })\\&h_j(\mathbf{q })=0,\quad \forall j \in (1..\ell )\\&g_i(\mathbf{q })\le 0,\quad \forall i \in (1..m). \end{aligned}$$

Let’s pretend, for a while, that the minima of ${\mathcal {A}}$ are different from zero. This hypothesis does not represent a restriction and the lack of it will be handled hereafter. Indeed, usually the cost function has some practical and physical meaning, therefore those minima reasonably lie within a known range. Assuming also that $\vartheta _0=1$, it is possible to write the following expression:

56 $$\begin{aligned} {\mathcal {F}}(\mathbf{q })={\mathcal {A}}(\mathbf{q })\prod _{i=1}^m\Theta \Big (-g_i(\mathbf{q })\Big )\prod _{j=1}^\ell \Theta \Big (h_j(\mathbf{q })\Big )\Theta \Big (-h_j(\mathbf{q })\Big ). \end{aligned}$$

As in Penalty Methods, the problem is reformulated as an unconstrained one by taking into account a so-called penalty function^26^. The proposed approach takes care of constraints by exploiting AND HSFG. Indeed, previous formulation is equivalent to:

57 $$\begin{aligned} {\mathcal {F}}(\mathbf{q })&={\mathcal {A}}(\mathbf{q })\Lambda \Big [\big \{N(g_i)\big \}\Big ]\Lambda \Big [\big \{h_j\big \}\Big ]\Lambda \Big [\big \{N(h_j)\big \}\Big ]\nonumber \\&= {\mathcal {A}}(\mathbf{q }){\overline{\Omega }}\Big [\big \{g_i\big \}\Big ]\Lambda \Big [\big \{h_j\big \}\Big ]{\overline{\Omega }}\Big [\big \{h_j\big \}\Big ]. \end{aligned}$$

As a matter of fact, the resultant function is zero across the whole space where they are not fulfilled, and it coincides with the cost function itself when they are satisfied. Moreover, each equality constraint is enabled through an AND HSFG having in input two face-to-face inequalities. The requirement $\vartheta _0=1$ which was stated before is herein clarified. With this assumption, gates return 1 when the weak inequalities $g_i(\mathbf{q })\le 0$ are satisfied. As a consequence, it also allows the equality constraints $h_j(\mathbf{q })$ to actually work. Otherwise, in both cases zero is provided. This behaviour reproduces the binary logic where the two levels are 0 and ${\mathcal {A}}(\mathbf{q })$ itself. Thus, the usage of the first level has to be denied. Indeed, if some minima are expected to be zeroes of ${\mathcal {A}}$, it is just needed to shift ${\mathcal {F}}$ sending ${\mathcal {A}}$ into ${\mathcal {A}}+c$. Clearly, the minimal obtained values have to be shifted back after they have been found.

The composition ${\mathcal {F}}$ cannot be globally convex because any of its second-order partial derivatives involves the second derivative of SF with respect to its argument. Therefore, convex optimization methods are not as well-suited as Heuristic SAs. However, these could need to move in a smooth manner between the two spaces (in which the constraints are satisfied or not). To this aim, once again, substitution (13) is necessary and hence SFGs have to be used instead of corresponding HSF forms. In this manner, an exponential slope is given to ${\mathcal {F}}$, and it will depend on $\alpha$. In particular, each equality constraint has to be multiplied by a factor 4 because the two $\sigma$ of which is composed of would give 1/2 each, when it is satisfied. The obtained function ${\mathcal {F}}$ and the optimization process are described by the pseudo-code in Algorithm 2.
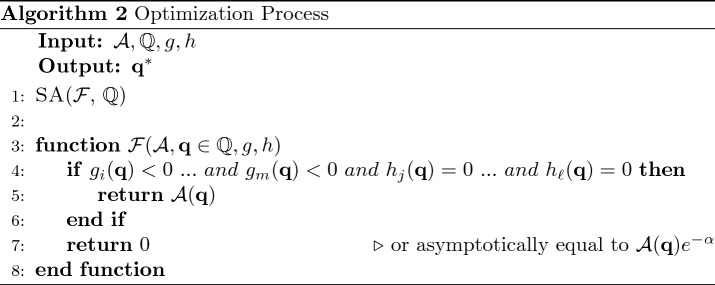


This method represents an alternative with respect to standard optimization, because the constraints are included in such a way that they are not constraints anymore, but a part of the whole function. Therefore, it is possible to avoid the check of the Karush–Kuhn–Tucker^27^ necessary conditions, because they rely on the construction of the Lagrangian while ${\mathcal {F}}$ is formally unconstrained. Thus, computation of weights concerning the classical inclusion of constraints is avoided. Most importantly, as a final remark, this method allows constraints also to be put in any other logical relation.

### Curves intersections

A useful geometrical application of proposed HSF Penalty Method (HSFPM) is to find how many and which intersections there are when two or more curves lie in the same locus. The methodology presented hereby is suitable in the case of two curves, but can be iterated in case of more. Consider $f_1$, $f_2$ and ${\mathbb {X}} = \{x_i \in {\mathbb {R}}\ \forall i:1..N_{MAX}\}$ and ${\mathbb {Y}} = \{y_i \in {\mathbb {R}}\ \forall i:1..N_{MAX}\}$, where $N_{MAX}$ is the maximum intersections number (see Bézout’s Theorem^28^). The optimization problem can be stated as follows

$$\begin{aligned} (P1)&\min _{{\mathbb {X}},{\mathbb {Y}}} \sum _{i=1}^{N_{MAX}} f_1(x_i,y_i)^2 + f_2(x_i,y_i)^2\ \mathbf{s}.t. \\&\left\Vert [x_k\ y_k]^T - [x_{j}\ y_{j}]^T\right\Vert \le \zeta \begin{array}{ll} &{}\forall k \in (1,N_{MAX}-1)\\ &{}\forall j \in (k+1,N_{MAX}),\\ \end{array} \end{aligned}$$

where $\zeta$ is a user-defined minimum distance to distinguish intersection points. This is a non-convex optimization problem that is hard to solve even with mathematical approximation. The technique proposed in this Section can be used to reformulate the problem as:

$$\begin{aligned} (P2) \max _{{\mathbb {X}},{\mathbb {Y}}} \sum _{i=1}^{N_{MAX}}\Theta \big (-f_i^*\big )\prod _{k<j}^{N_{MAX}}{\mathcal {J}}_{kj}, \end{aligned}$$

where

$$\begin{aligned}&{\mathcal {J}}_{kj} = \Theta \Big (\left\Vert [x_k\ y_k]^T - [x_j\ y_j]^T\right\Vert - \zeta \Big ),\\&f_i^* = f_1^2(x_i,y_i) + f_2^2(x_i,y_i). \end{aligned}$$

A SA can be used to solve this problem.

Basically, every intersection point corresponds to a maxima of the objective function. Moreover, a $\Theta (\cdot )$ has been introduced in the objective function derived from (*P*1) in order to assure that local maxima are avoided in (*P*2), and the function value will now be equal to the number of intersections which the algorithm finds. In fact, every other point which is not a real intersection would not change the discrete function value. However, as it was previously remarked, $\Theta (\cdot )$ has to be substituted with $\sigma (\alpha \cdot )$ in order for the algorithm to decently move in the solution domain and $\alpha$ has to be relatively small. As a consequence, the points in excess would increase the function value in an undefined manner. So, it is necessary to introduce a new integer parameter *N* which counts the real intersections. As the previous sum and products now extend up to *N*, one more addendum *P* is introduced as a penalty cost for having increased *N*, but in such a way that it must be convenient to pay it if a true intersection is found. To this aim it is necessary to also multiply the objective function for a weight *W*, such that:

58 $$\begin{aligned} W\sigma (-\alpha \zeta ) < P . \end{aligned}$$

The worst case scenario is when the algorithm tries to count a point at a distance $\zeta$ from a true intersection as if it was a real one. The requirement in Eq. (58) prevents such unwanted behaviour, as it imposes that when a fake intersection is found the reward gained has to be lower than inflicted penalty. For instance, to achieve this goal, (*P*2) can be reformulated as:

$$\begin{aligned} (P3) \max _{{\mathbb {X}},{\mathbb {Y}},N} 2N_{MAX}\sum _{i=1}^{N}\sigma \big (-\alpha f^*\big )\prod _{k<j}^{N}{\mathcal {J}}_{kj} - (N_{MAX} - N). \end{aligned}$$

where ${\mathcal {J}}_{kj}$ is now made with the substitution (13). Despite aforementioned solution, others can be investigated.

## Numerical evaluation

In this Section, theoretical results discussed in past Sections are evaluated. In particular, a wide simulation campaign has been carried out to analyze each possible application derived from discussed previous notions. The adopted hardware consists of a desktop computer equipped with Intel i7 970 and 16GB RAM. In all the optimization-based applications, GA implemented in MATLAB R2020a, is employed as SA of reference. For the sake of generality, measurement units are simplified to pure numbers.

### Volumes measure error analysis

In order to evaluate results obtained in “Computation of volume measure covered by intersecting geometrical loci”, a reference scenario is taken into account. Given the general equation of a circle

59 $$\begin{aligned} {\mathcal {C}}(x_0,y_0,r): (x-x_0)^2+(y-y_0)^2 - r^2 = 0, \end{aligned}$$

the aim is to compute the union area of ${\mathcal {C}}_1 = {\mathcal {C}}(-1,0,1)$ and ${\mathcal {C}}_2 = {\mathcal {C}}(1,0,1)$, which are external tangent, placed in a mesh 4x2. This task can be done exploiting Eq. (40):

60 $$\begin{aligned}&\int _{\mathcal {M}} \Omega \Big [N({\mathcal {C}}_1),N({\mathcal {C}}_2)\Big ](\mathbf{x }) d\mathbf{x } = \end{aligned}$$

61 $$\begin{aligned} &=\int _{-1}^{1}\int _{-2}^{2} \Theta \Big ( \Theta \big (-((x+1)^2+y^2-1)\big ) \nonumber \\&\quad +\Theta \big (-((x-1)^2+y^2-1)\big ) - 0.1\Big ) dxdy. \end{aligned}$$

First, the integral has been calculated in MATLAB through the Riemann Summation, with a mesh precision $\rho$ = 10$^{-4}$. The exact result of the area should be 2$\pi$R$^2 \simeq$6.28318 but, due to the precision used, the experimental one is $\simeq$6.28313. This evaluation considers HSFGs that have a discontinuity in 0 that does not represent a problem as stated in Remark 2. However, when SFGs are used, $\alpha$ has an non-negligible impact on the error as discussed in “Computation of volume measure covered by intersecting geometrical loci”.

**Figure 4:**
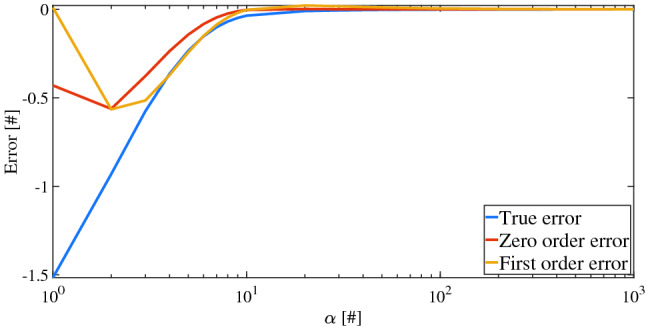
Comparison between experimental error and theoretical one in a reference scenario.

As expected Fig. 4 shows that, fixed the mesh precision, the error of $\sigma (\cdot )$, with respect to $\Theta (\cdot )$, decreases as $\alpha$ increases. Moreover, the first two orders shown demonstrate that (i) the high-order derivatives accounts for lower $\alpha$ error (ii) for $\alpha \ge 10$ the error is negligible.

### Custom volume measure

**Figure 5:**
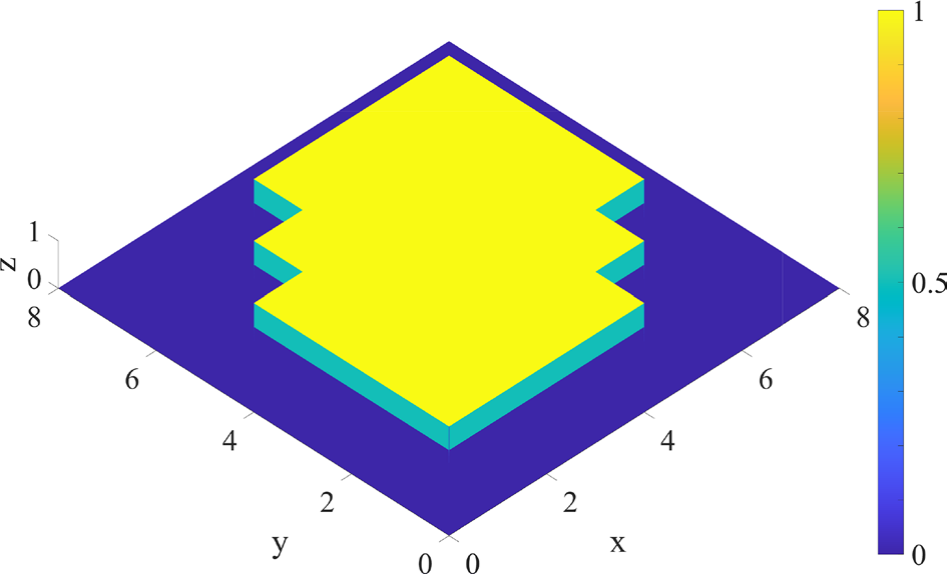
Intersection of $${\mathcal {S}}_1$$, $${\mathcal {S}}_2$$, $${\mathcal {S}}_3$$.

Thanks to this analysis and results obtained in “Custom D-dimensional volumes”, it is possible to solve problem in the scenario proposed in “Computation of volume measure covered by intersecting geometrical loci”. First, the function that describes the Square #1, namely ${\mathcal {S}}_1$, centered in $[3\ 3]^T$, can be easily derived from Eq. (50):

62 $$\begin{aligned} {\mathcal {S}}_1 = \Lambda _\alpha \Big [x-1,-(x-5),y-1,-(y-5)\Big ]. \end{aligned}$$

Similarly, it is possible to build the other two squares ${\mathcal {S}}_2$ and ${\mathcal {S}}_3$. The integrand of Eq. (40) is shown in Fig. 5. As previously mentioned, the edges of the volume have a value of 1/2 that is exactly $\sigma (0)$. Finally, exploiting Eq. (40), with $\rho$ = 10$^{-3}$, $\alpha$ = 10$^{3}$ and a mesh 8x8, the calculated area is 29.99998.

### HSF penalty method

To probe results of “HSF Penalty Method” two simulations of different complexity have been done.

The first involves two fixed circles ${\mathcal {C}}_1 = {\mathcal {C}}(-2,0,1)$ and ${\mathcal {C}}_2 = {\mathcal {C}}(2,0,1)$ placed in a 8x8 mesh. A third one, defined as ${\mathcal {C}}_3 = {\mathcal {C}}(x,y,1)$ has to be positioned in order to maximize the total covered area. Taking advantage of Eq. (40) and (13), ${\mathcal {V}}_\sigma$ is defined as the SF variant of the Heaviside’s one.

**Figure 6:**
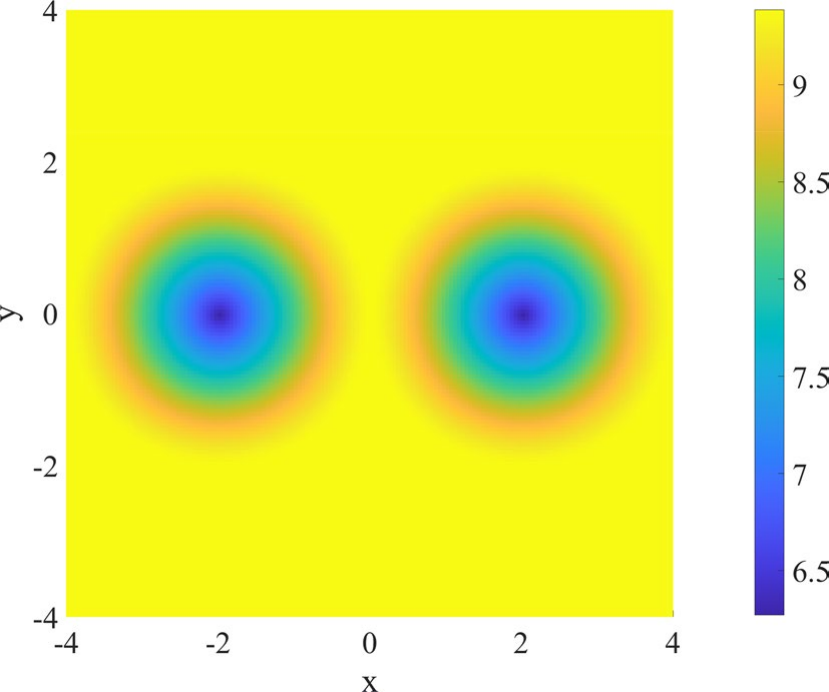
Total covered area as a function of ${\mathcal {C}}_3$ center.

The task is accomplished by the objective function:

63 $$\begin{aligned} {\mathcal {V}}_\sigma \Big [\{{\mathcal {C}}_n\}\Big ]\quad \forall n \in (1,3), \end{aligned}$$

where $\alpha$ = 10$^3$ and $\rho$ = 5$\times$10$^{-2}$. The locus in which the center $[x \ y]^T$ of ${\mathcal {C}}_3$ has to be placed to maximize the total covered area is shown in Fig. 6. As expected, the center should not be in proximity of the two pre-positioned circles.

The second experiment emulates a constrained simple scenario in which a drone, equipped with a camera, has to take off and land in the same spot. Throughout the mission, the drone has to pursue a trajectory and scan the maximum possible ground area which is composed of camera shoots over the (*x*, *y*)-plane. It is assumed that the drone cannot exceed a maximum speed, it flies at constant altitude and the ratio of acquired images is constant and set to unity.

From an optimization point of view, the problem can be stated as follows:

$$\begin{aligned}{}&\max _{{\mathbb {X}},{\mathbb {Y}}} {\mathcal {V}}_\sigma \Big [\{{\mathcal {C}}_n\}\Big ]\ \mathbf{s}.t. \\&\left\Vert [x_k\ y_k]^T - [x_{j}\ y_{j}]^T\right\Vert \le v_{MAX} \begin{array}{ll} &{}\forall k \in (1,N-1)\\ &{}\forall j \in (k+1,N)\\ \end{array},\\&x_1 = y_1 = x_{N} = y_{N} , \end{aligned}$$

where *N* is the number of circles ${\mathcal {C}}_n$, whereas ${\mathbb {X}}$ and ${\mathbb {Y}}$ are the sets of centers $n:2..N-1$ coordinates of which trajectory is made of.

The start/end points are settled to $[-2 \ -2]^T$. Since the acquisition ratio is set to unity, the speed *v* of the drone corresponds to the distance that is travelled between two consequent points.

**Figure 7:**
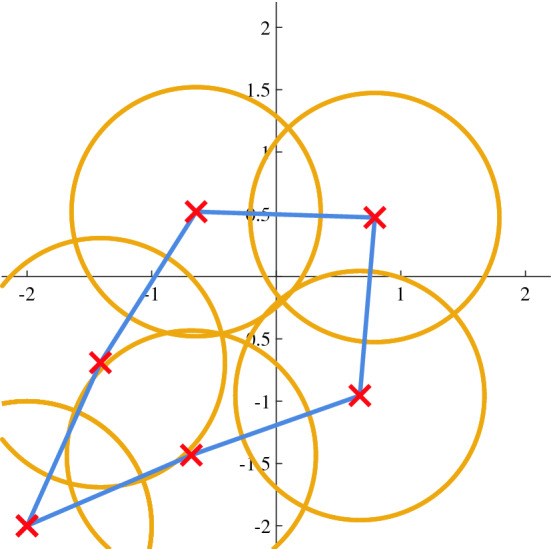
Optimized constrained drone trajectory.

Motivated by Eq. (56), it is possible to reformulate the problem stated above as:

$$\begin{aligned} \max _{{\mathbb {X}},{\mathbb {Y}}} {\mathcal {V}}_\sigma \Big [ \{{\mathcal {C}}_n\}\Big ]\prod _{k<j}^{N}{\mathcal {J}}_{kj}, \end{aligned}$$

where

64 $$\begin{aligned}&{\mathcal {J}}_{kj} = \sigma \bigg (\alpha \Big (\left\Vert [x_k\ y_k]^T - [x_j\ y_j]^T\right\Vert - v_{MAX}\Big )\bigg ). \end{aligned}$$

The test has been run in a 4x4 mesh with precision $\rho$ = 10$^{-2}$, $N=7$ circles, $v_{MAX}$ = 1.5 and $\alpha$ = 10$^2$.

Results of the solution of this problem are shown in Fig. 7. Based on solution found by the algorithm, the area covered is $\sim$11.5. Due to the applied constraint, projected areas are overlapped and cut by the mesh borders. It also results that the adopted speed in each segment is about the maximum possible because, indeed, it minimizes the overlaps.

### Custom D-dimentional volumes

**Figure 8:**
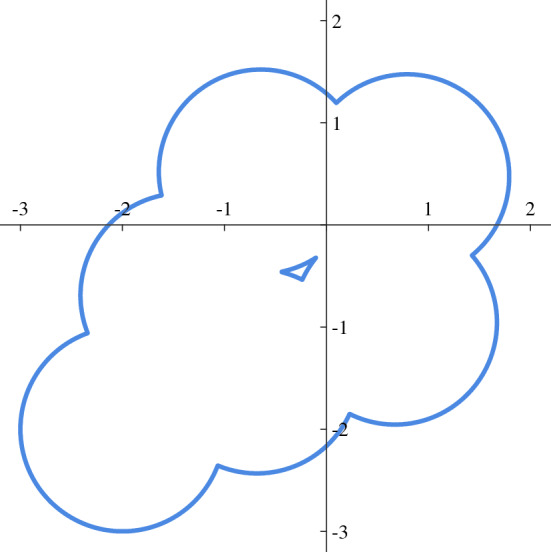
Border of area covered by drone’s trajectory.

In the previous Subsection an interesting pattern has been obtained in terms of covered area. The associated ${\mathcal {X}}_\sigma$ can be useful to test results achieved in “Custom D-dimensional borders”. The outcome of this simulation is shown in Fig. 8, using a 5x5 mesh, $\rho$ = 10$^{-3}$ and $\alpha$ = 10$^3$. It is worth noting that, even if holes are present in the union of volumes, this is not a problem and a union border $\Gamma$ can always be realized.

### Curves intersections

**Figure 9:**
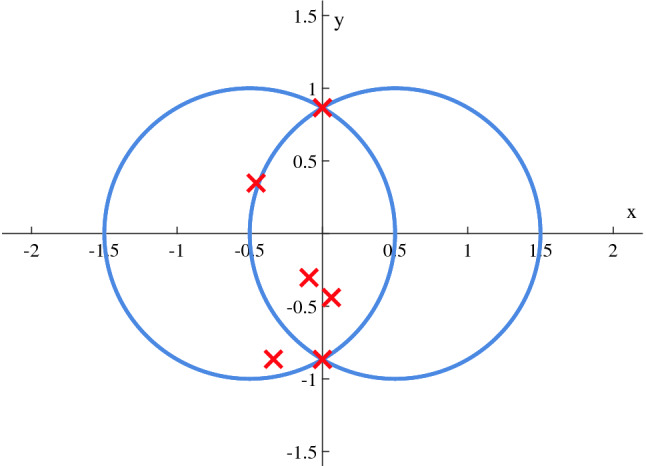
Intersection points of two circles found via GA.

**Figure 10:**
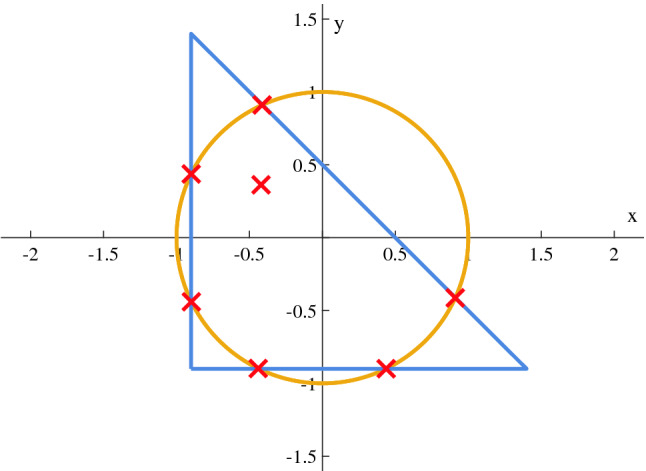
Intersection points of a circle and a triangle found via GA.

Another aspect of optimization, discussed in “Curves intersections”, is intersection counting and discovering. Two scenarios have been settled. In the first, two circles ${\mathcal {C}}_1 = {\mathcal {C}}(-0.5,0,1)$ and ${\mathcal {C}}_2 = {\mathcal {C}}(0.5,0,1)$ are considered. In the second, a circle ${\mathcal {C}}_3 = {\mathcal {C}}(0,0,1)$ and a triangle ${\mathcal {T}}$ of vertices $(-0.9,1.4)$, $(1.4,-0.9)$ and $(-0.9,-0.9)$ are studied. Thanks to formulation in (*P*3), results of these cases are shown in Figs. 9 and 10 where $N_{MAX}$ = 7 and $\alpha$ = 10.

In both cases, the returned vector of solution contains the ordered set of information being: the number *N* of actual intersections (2 and 6 respectively) followed by the related coordinates and then the fake $N_{MAX}-N$ ones. Experimentally, it is important to mention that as the complexity of the involved scenario increases, the research of intersections gets harder in the sense that some runs may not found all of them. In particular, when two or more intersection are close, distinguishing becomes an hard process that could be overcome as fine-tolerance algorithms are used. Another essential note is correlated with custom volumes and, specifically, for what concern oblique segments. On a trial basis, outcomes of simulations confirm that $\alpha$ related to these pieces of volumes should be set not greater than 10 with the aim of speed up the SA operations.

## Conclusion

The present work discussed a mathematical characterization of binary logic granted by the HSF. Then, an analytical representation through logistic function has been studied. Furthermore, the existence of a correlation between the conceived mathematical structure and a finite-time program has been proved.

Based on these concepts, different geometrical applications and a Penalty Method for constrained optimization problems has been deeply investigated. First, the measure of binary operations among D-volumes is presented. These can also be designed in a custom manner so that a compact region is encompassed by a set of loci. Hence, it is even possible to obtain the border analytical expression of the desired D-volume. Finally, a novel Penalty Method has been realized allowing the reformulation of a constrained non-convex optimization problem in an unconstrained one.

A wide simulation campaign demonstrated the effectiveness of proposed methodologies which can be employable in many engineering areas such as computer vision and graphics, medical imaging, robotics and automation.

However, several aspects can be further investigated. First of all, a solid correspondence between control structures and proposed HSFGs/SFGs will be addressed. Then, a method to compute the border length of a custom volume will be probed. Current formulation to find intersections could also be improved. Finally, the proposed Penalty Method will be tested with more SAs and compared with other solutions, to settle a proper benchmark.

## Supplementary Information

Supplementary Information.
