# Supplementary material for: Representing logic gates over Euclidean space via heaviside step function
Source: Sci Rep. 2022 May 14;12:8009. doi: 10.1038/s41598-022-11941-y (PMC9107489; doi:10.1038/s41598-022-11941-y)
Supplement: Supplementary file 1 — Supplementary Information. [file 41598_2022_11941_MOESM1_ESM.pdf]

# Representing Logic Gates over Euclidean Space via Heaviside Step Function

Giovanni Iacovelli[a,b,], Claudio Iacovelli[c]

[a]Department of Electrical and Information Engineering (DEI), Politecnico di Bari, 70126 Bari, Italy
[b]Consorzio Nazionale Interuniversitario per le Telecomunicazioni (CNIT), 43124 Parma, Italy
[c]Institut de Ciències Fotòniques (ICFO), The Barcelona Institute of Science and Technology, 08860 Castelldefels, Spain

## Appendix A. Proof Theorem 2

Define $\mathcal{H}^1 = \Theta \circ g_1 \circ \vec{\Theta}_0 = \Theta \circ h_1$ and its domain as $dom(\mathcal{H}^1) = \{\mathbf{x} \in \mathbb{R}^D \ni' h_1 \neq 0\}$. Suppose there exists a $k$ such that $h_1\big|_{Q_k} = c_k \neq 0$. Now let such $k$ be fixed, and verify that:

$$\begin{aligned}&\forall \epsilon > 0 \ \exists\ \overline{\alpha}_k > 0 \ni' \ \forall \alpha \geq \overline{\alpha}_k :\\ &\big|\mathcal{H}^1(\mathbf{x}) - \mathcal{H}^1_\alpha(\mathbf{x})\big| = \big|\sigma_\alpha\big(h_{1,\alpha}(\mathbf{x})\big) - \Theta\big(h_1(\mathbf{x})\big)\big| < \epsilon \ \forall \mathbf{x} \in X_{t,k},\end{aligned}$$

where $\overline{\alpha}_k$ depends on $Q_k$. The above can be demonstrated by adding and subtracting $\sigma_\alpha\big(h_1(\mathbf{x})\big)$. Hence, $\forall \mathbf{x} \in X_{t,k}$, it results that:

$$\begin{aligned}\big|\mathcal{H}^1(\mathbf{x}) - \mathcal{H}^1_\alpha(\mathbf{x})\big| &\leq \big|\sigma_\alpha\big(h_{1,\alpha}(\mathbf{x})\big) - \sigma_\alpha\big(h_1(\mathbf{x})\big)\big| + \\ &+ \big|\sigma_\alpha\big(h_1(\mathbf{x})\big) - \Theta\big(h_1(\mathbf{x})\big)\big|.\end{aligned}$$

First, fix $\epsilon > 0$ and check the second addenda of the RHS. As previously stated, $\forall \mathbf{x} \in X_{t,k} : h_1(\mathbf{x}) = c_k \neq 0$ that implies

$$\sigma_\alpha\big(h_1(\mathbf{x})\big) = \sigma_\alpha(c_k) \quad \text{and} \quad \Theta\big(h_1(\mathbf{x})\big) = \Theta(c_k).$$

Since $c_k \neq 0$, thanks to Theorem 5 (see **Appendix B**) $\sigma_\alpha(c_k) \to \Theta(c_k)$. Hence, in correspondence of $\epsilon/2$

$$\exists\ \overline{\alpha}_{1,k} \ni' \ \forall \alpha \geq \overline{\alpha}_{1,k} : \ \big|\sigma_\alpha(c_k) - \Theta(c_k)\big| < \epsilon/2.$$

and the inequality is equivalent to

$$\big|\sigma_\alpha\big(h_1(\mathbf{x})\big) - \Theta\big(h_1(\mathbf{x})\big)\big| < \epsilon/2 \quad \forall \mathbf{x} \in X_{t,k}. \tag{A.1}$$

Then, check the first addenda of the RHS. Define $c := \frac{|c_k|}{2}$. Now, suppose that $c_k > 0$: recalling that $h_{1,\alpha} \to c_k$ uniformly in $X_{t,k}$,

$$\begin{aligned}&\exists\ \overline{\alpha}_k^+ \ni' \forall \alpha \geq \overline{\alpha}_k^+, \forall \mathbf{x} \in X_{t,k} : h_{1,\alpha}(\mathbf{x}) \geq c_k/2 = c\\ &\implies \forall \alpha \geq \overline{\alpha}_k^+, \forall \mathbf{x} \in X_{t,k} : \quad \begin{matrix} h_{1,\alpha}(\mathbf{x}) \geq c \\ h_1(\mathbf{x}) = c_k \geq c. \end{matrix}\end{aligned}$$

Recalling Lemma 1 (see **Appendix C**) with $x = h_{1,\alpha}(\mathbf{x})$ and $y = h_1(\mathbf{x})$

$$\left|\sigma_\alpha\big(h_{1,\alpha}(\mathbf{x})\big) - \sigma_\alpha\big(h_1(\mathbf{x})\big)\right| \leq L_c\left|h_{1,\alpha}(\mathbf{x}) - h_1(\mathbf{x})\right|. \tag{A.2}$$

Instead, if $c_k < 0$:

$$\begin{aligned} &\exists\ \overline{\alpha}_k^- \ni' \forall\alpha \geq \overline{\alpha}_k^-, \forall\mathbf{x} \in X_{t,k} : h_{1,\alpha}(\mathbf{x}) \leq c_k/2 = -c \\ &\implies \forall\alpha \geq \overline{\alpha}_k^-, \forall\mathbf{x} \in X_{t,k} : \quad \begin{matrix} h_{1,\alpha}(\mathbf{x}) \leq -c \\ h_1(\mathbf{x}) = c_k \leq -c. \end{matrix} \end{aligned}$$

Lemma 1 holds in this case as it was for (A.2). Thus, taking $\overline{\alpha}_{2,k} := \max\{\overline{\alpha}_k^+, \overline{\alpha}_k^-\}$,

$$\left|\sigma_\alpha\big(h_{1,\alpha}(\mathbf{x})\big) - \sigma_\alpha\big(h_1(\mathbf{x})\big)\right| \leq L_c\left|h_{1,\alpha}(\mathbf{x}) - h_1(\mathbf{x})\right| \quad \begin{matrix} \forall\alpha \geq \overline{\alpha}_{2,k} \\ \forall\mathbf{x} \in X_{t,k}. \end{matrix}$$

As a consequence of Theorem 5, in correspondence of $\frac{\epsilon}{2L_c}$:

$$\exists\ \overline{\alpha}_{3,k} \ni'\ \forall\alpha \geq \overline{\alpha}_{3,k} :\ \left|h_{1,\alpha}(\mathbf{x}) - h_1(\mathbf{x})\right| < \frac{\epsilon}{2L_c} \quad \forall\mathbf{x} \in X_{t,k}. \tag{A.3}$$

Therefore, for $\alpha \geq \max\{\overline{\alpha}_{2,k}, \overline{\alpha}_{3,k}\}$:

$$\left|\sigma_\alpha\big(h_{1,\alpha}(\mathbf{x})\big) - \sigma_\alpha\big(h_1(\mathbf{x})\big)\right| \leq \epsilon/2 \quad \forall\mathbf{x} \in X_{t,k}. \tag{A.4}$$

Finally, for $\overline{\alpha}_k \geq \max\{\overline{\alpha}_{1,k}, \overline{\alpha}_{2,k}, \overline{\alpha}_{3,k}\}$, (A.1) and (A.4) holds at same time and hence:

$$\left|\mathcal{H}^1_\alpha(\mathbf{x}) - \mathcal{H}^1(\mathbf{x})\right| \leq \epsilon \quad \forall\mathbf{x} \in X_{t,k}. \tag{A.5}$$

This demonstration holds in each $X_{t,k}$, $\forall k \in J_1$, with $J_1 = \{k : 1...2^D \ni' h_1\big|_{Q_k} = c_k \neq 0\}$. Indeed, taking $\widetilde{\alpha} = \max_{k\in J_1}\{\overline{\alpha}_k\}$, the convergence is proved in $\widetilde{X}_1 := \bigcup_{k\in J_1} X_{t,k}$. Further, the discussed results can be easily extended whatever the dimensionality of $h_1$ is, i.e. $\vec{h}_1 = \vec{g}_1 \circ \vec{\Theta}_0$. In fact, with dutiful dimension-related considerations and taking $\widetilde{\alpha} = \max_{k\in J_1}\{\overline{\alpha}_k\}$, it can be seen that $\vec{h}_1 \to \vec{h}_{1,\alpha}$ in $\widetilde{X}$, where $J_1 = \{k : 1...2^D \ni' \vec{h}_1\big|_{Q_k} = \vec{c}_{1,k}, c^u_{1,k} \neq 0\ \forall u : 1...N_1\}$. Finally, it can be generalized for every rank, i.e. number of compositions, with $\widetilde{\alpha}$ related to $J = \{k : 1...2^D \ni' \vec{h}_r\big|_{Q_k} = \vec{c}_{r,k}, c^u_{r,k} \neq 0\ \forall u : 1...N_{r+1}, \forall r : 1...R\}$.

## Appendix B. Convergence of the first composition

**Theorem 5.** *Let $\vec{g}_1$ be a uniformly continuous function, $\vec{h}_{1,\alpha} = \vec{g}_1 \circ \vec{\sigma}_{0,\alpha}$ and $\vec{h}_1 = \vec{g}_1 \circ \vec{\Theta}_0$. Then, $\vec{h}_{1,\alpha}$ converges in $X_t$ uniformly to $\vec{h}_1$ for $\alpha \to \infty$.*

*Proof.* The family of functions $\{\delta_\alpha | \alpha > 0\}$ pointwise converges in $\mathbb{R}$ for $\alpha \to +\infty$ to

$$\delta_\infty(x) = \begin{cases} 0 & \text{if } x \neq 0 \\ \vartheta_0 - \frac{1}{2} & \text{if } x = 0. \end{cases} \tag{B.1}$$

Therefore, it means that $\{\sigma(\alpha\cdot)\}_\alpha$ pointwise converges in $\mathbb{R}^*$ to $\Theta(\cdot)$ and in $\mathbb{R}$ to $\Theta(\cdot)$ only if $\vartheta_0 = 1/2$. Instead $\{\delta_\alpha\}_\alpha$ uniformly converges in $A_t = ]-\infty, -t] \cup [t, +\infty[\ \forall t > 0$ for $\alpha \to +\infty$ to $\delta_\infty$, meaning that uniform convergence of $\{\sigma(\alpha\cdot)\}_\alpha$ to $\Theta(\cdot)$ is only achieved in the same interval. Therefore, $\vec{\sigma}_{0,\alpha}$ converges to $\vec{\Theta}_0$ in a component-wise manner and with the same caveats as before. Hence, the convergence of the whole vector function can be established with the metric $d := d_{\mathbb{R}^\nu}$ in $A_t^D = X_t$, with $\nu$ being the dimensionality of the euclidean space. The fact that $\vec{g}_1$ is a uniform continuous function means that

$$\begin{aligned}&\forall \epsilon > 0\ \exists \delta > 0 \ni' \ \forall \xi, \eta \in \widetilde{\mathcal{B}}^D, d(\xi, \eta) < \delta : \\ &d(\vec{g}_1(\xi), \vec{g}_1(\eta)) < \epsilon.\end{aligned}$$

Fix $\epsilon > 0$, then $\delta > 0$ is fixed. Since $\vec{\sigma}_{0,\alpha}$ converges to $\vec{\Theta}_0$ uniformly in $X_t$, in correspondence of $\delta > 0$:

$$\exists\ \overline{\alpha} \ni' \forall \alpha \geq \overline{\alpha} :\ d\left(\vec{\sigma}_{0,\alpha}(\mathbf{x}), \vec{\Theta}_0(\mathbf{x})\right) < \delta\ \forall \mathbf{x} \in X_t.$$

Therefore, setting $\xi = \vec{\sigma}_{0,\alpha}(\mathbf{x})$ and $\eta = \vec{\Theta}_0(\mathbf{x})$, it is obtained $d(\vec{g}_1(\vec{\sigma}_{0,\alpha}(\mathbf{x})), \vec{g}_1(\vec{\Theta}_0(\mathbf{x}))) < \epsilon$ which is:

$$d\left(\vec{h}_{1,\alpha}(\mathbf{x}), \vec{h}_1(\mathbf{x})\right) < \epsilon.$$

□

## Appendix C. $\{\sigma_\alpha\}_{\alpha>0}$ is equi-Lipschitz

**Lemma 1.** *Let $c \in \mathbb{R}_+^*$, and define $A_c = ]-\infty, -c] \cup [+c, +\infty[$. Then, $\{\sigma_\alpha\}_{\alpha>0}$ is an equi-Lipschitz family in $A_c$, that is:*

$$\begin{aligned}&\exists L_c > 0 \ni' \forall x, y \in A_c : \\ &\left|\sigma_\alpha(x) - \sigma_\alpha(y)\right| \leq L_c|x - y|\ \forall \alpha > 0.\end{aligned}$$

*Proof.* Given $c > 0$, set $L_c := \max_{\alpha\geq 0} \sigma'_\alpha(c)$. Reminding that (i) $\sigma'_\alpha(c) \to 0$ for $\alpha \to +\infty$ (ii) it is non-negative $\forall \alpha > 0$ (iii) it is 0 for $\alpha = 0$ and (iv) it is a continuous function then for the generalized Weierstrass Theorem $\sigma_\alpha(c)$ as a function of $\alpha$ must have a positive maxima. This guarantees that $L_c > 0$. Now, consider $x, y \in ]-\infty, -c]$ and $\alpha > 0$. Recalling Lagrange Theorem, there exists $\xi \in ]x, y[$ such that:

$$\sigma_\alpha(x) - \sigma_\alpha(y) = \sigma'_\alpha(\xi)(x - y). \tag{C.1}$$

Since $x, y \in ]-\infty, -c] \implies \xi \in ]-\infty, -c]$, hence $\sigma'_\alpha(\xi) \leq \sigma'_\alpha(c) \leq L_c$. Therefore, (C.1) can be rewritten as

$$\left|\sigma_\alpha(x) - \sigma_\alpha(y)\right| \leq L_c|x - y|, \tag{C.2}$$

and this has to hold $\forall \alpha > 0$ because of the definition of $L_c$. This rationale is analogous for $[+c, +\infty[$. □
